# Transcriptome analysis of mRNA and miRNA in the development of LeiZhou goat muscles

**DOI:** 10.1038/s41598-024-60521-9

**Published:** 2024-04-29

**Authors:** Junjie Fu, Jie Liu, Xian Zou, Ming Deng, Guangbin Liu, Baoli Sun, Yongqing Guo, Dewu Liu, Yaokun Li

**Affiliations:** 1 College of Animal Science, South China Agricultural University, Guangzhou, 510642 China; 2 State Key Laboratory of Livestock and Poultry Breeding, Guangdong Key Laboratory of Animal Breeding and Nutrition, Institute of Animal Science, Guangdong Academy of Agricultural Sciences, Guangzhou, 510640 China; 3 National Local Joint Engineering Research Center of Livestock and Poultry, South China Agricultural University, Guangzhou, 510642 China

**Keywords:** Sequencing, Computational biology and bioinformatics

## Abstract

The progression of muscle development is a pivotal aspect of animal ontogenesis, where miRNA and mRNA exert substantial influence as prominent players. It is important to understand the molecular mechanisms involved in skeletal muscle development to enhance the quality and yield of meat produced by Leizhou goats. We employed RNA sequencing (RNA-SEQ) technology to generate miRNA-mRNA profiles in Leizhou goats, capturing their developmental progression at 0, 3, and 6 months of age. A total of 977 mRNAs and 174 miRNAs were found to be differentially expressed based on our analysis. Metabolic pathways, calcium signaling pathways, and amino acid synthesis and metabolism were found to be significantly enriched among the differentially expressed mRNA in the enrichment analysis. Meanwhile, we found that among these differentially expressed mRNA, some may be related to muscle development, such as MYL10, RYR3, and CSRP3. Additionally,, we identified five muscle-specific miRNAs (miR-127-3p, miR-133a-3p, miR-193b-3p, miR-365-3p, and miR-381) that consistently exhibited high expression levels across all three stages. These miRNAs work with their target genes (FHL3, SESN1, PACSIN3, LMCD1) to regulate muscle development. Taken together, our findings suggest that several miRNAs and mRNAs are involved in regulating muscle development and cell growth in goats. By uncovering the molecular mechanisms involved in muscle growth and development, these findings contribute valuable knowledge that can inform breeding strategies aimed at enhancing meat yield and quality in Leizhou goats.

## Introduction

In recent years, China's goat industry has shown significant growth, particularly in goat meat production. Among low-fat meat options, goat meat is highly favored for its unique flavor, palatability, and relatively lean nature when compared to other red meats^1^. Skeletal muscle makes up approximately 40% of a goat's body weight^2^, highlighting its significance as a crucial component of mutton. Enhancing the growth and development of skeletal muscle is crucial for animal agriculture as it provides a source of meat for human consumption. Consequently, studying the differentiation and development of skeletal muscle in goats is crucial for the advancement of modern agriculture.

Before birth, skeletal muscle growth and development primarily occur through an increase in muscle fibers, resulting in skeletal muscle hypertrophy^3^. After birth, skeletal muscle hypertrophy is achieved through the enlargement of existing muscle fibers and the activation of a population of muscle stem cells. Therefore, postnatal skeletal muscle growth is governed by distinct mechanisms from embryonic muscle development^4^. Research has shown that myostatin is critical for fetal muscle development but is not required for postnatal skeletal muscle growth. Instead, postnatal skeletal muscle growth may be more dependent on Ca2 + signaling and Mef2 proteins, rather than myogenic basic helix-loop-helix (bHLH) factors^5^. Ca2 + signaling can facilitate signal transduction during neonatal muscle development^6^, while Mef2 proteins may serve as a major regulator of skeletal muscle growth, playing a crucial role in postnatal skeletal muscle growth as well^5^. In summary, while embryonic and postnatal skeletal muscle growth share some common features, the underlying mechanisms controlling these processes are distinct. Postnatal skeletal muscle growth is primarily regulated by Ca2 + signaling and Mef2 proteins, with myostatin playing a minor role.

In the past, people who wanted to improve traits such as skeletal muscle in goats to increase meat yield could often only breed strong individuals through individual or group selection, but this approach has the problem of low efficiency and instability. Now it is possible to select goats with superior genes through genetic screening, which greatly accelerates this process, thus advancing the development of the industry. In the research investigations in this paper, we observed that skeletal muscle is regulated by many mRNAs and miRNAs. MiRNAs, which are short non-coding RNA molecules approximately 19–23 nucleotides in length, regulate gene expression by interacting with target mRNA^7^ and play crucial roles in various biological processes, such as cell proliferation^8^, differentiation^9^, and apoptosis^10^, as well as immune response^11^, tumors^12^, and neurological development^13^. In addition to these functions, miRNAs also play an essential role in muscle development and differentiation. Inhibition of miRNA expression can lead to increased expression of target gene proteins and mRNA. Several studies have investigated the specific roles of miRNAs in muscle development and differentiation. McCarthy JJ et al.^14^ found that the predicted target mRNAs of miR-1 and miR-133a, such as c-Met, HGF, IGF-1, SRF, and LIF, are all known to be important genes in muscle growth. Decreased miRNA expression during overload may help promote the expression of these genes. IGF-1, in particular, has a well-documented role in skeletal muscle hypertrophy. Chen JF et al.^15^ observed that both miR-1 and miR-206 were sharply downregulated in injured skeletal muscle and inhibited Pax7 protein expression in differentiated muscle progenitor cells. This suggests that miR-1 and miR-206 regulate the transition from proliferation to differentiation in skeletal muscle satellite cells. Liu N et al.^16^ found that miR-206 promotes satellite cell differentiation and fusion into muscle fibers by inhibiting the accumulation of negative regulatory factors of myogenesis. Zhang Z et al.^17^ discovered that the content of miR-145-5p was moderately high in the muscle of lamb compared to goat fetus muscle. They also observed that miR-145-5p inhibited the development and apoptosis of goat myoblasts by suppressing USP13. In the functional and regulatory mechanisms of goat muscle proliferation and development, Ling YH et al.^18^ found that Pax3 expression could maintain myogenic progenitor cells in an undifferentiated state. They also discovered that the degradation of Pax3 was essential for myogenic differentiation. Furthermore, miR-27b was identified as a regulator of myogenic proliferation and differentiation by targeting goat Pax3. IRS1, a target gene for miR-487b-3p, coordinates skeletal muscle growth and metabolism through the Akt and AMPK pathways, and induces myoblast proliferation and myotube hypertrophy by increasing phosphorylated Akt levels^19^. Therefore, miRNAs play a crucial role in muscle growth and development by regulating genes related to skeletal muscle cell proliferation, differentiation, apoptosis, and other functions. The discovery of additional miRNAs and mRNAs associated with muscle growth and development will enhance our understanding of the mechanisms involved in the growth and development process. It will also provide a theoretical foundation for elucidating the molecular mechanisms of skeletal muscle development in Leizhou goats.

RNA sequencing (RNA-seq) is a powerful high-throughput technique for quantifying and analyzing transcripts. By using RNA-seq, researchers can measure mRNA expression levels, detect gene exons, variable splice isoforms, and identify novel genes on a genome-wide scale.

Leizhou goat is a goat breed unique to the city of Leizhou in Guangdong Province, China. It is known for its adaptability, hunger, cold tolerance, and disease resistance. The Leizhou goat is highly adaptable to foraging and can effectively utilize herbaceous plants and low-quality forage. However, the breed has significantly deteriorated due to a chronic lack of effective conservation measures, repeated inbreeding, and inappropriate breeding management procedures. In goat breeding, lambs are typically weaned at around three months of age and then fattened between 6–8 months. Therefore, we utilized RNA-Seq technology to conduct transcriptome profiling of the longest dorsal muscles of Leizhou goats at 0, 3, and 6 months postnatally. Through this analysis, we identified relevant mRNAs and miRNAs that influence muscle development and differentiation, along with their target genes. We conducted functional enrichment analysis of the differentially expressed genes. This analysis offers a theoretical foundation for further understanding the molecular mechanisms involved in skeletal muscle development in Leizhou goats. It also presents novel insights for molecular breeding and the advancement of Leizhou goats.

## Methods

### Sample collection

The goats used in this experiment were from a goat breeding facility in Leizhou City, Guangdong Province. Nine healthy male Leizhou goats were divided into three groups under the same feeding management and nutritional conditions. The animals were slaughtered at 0, 3, and 6 months of age, respectively. Immediately after slaughter, the longest dorsal muscle tissue samples were removed, packed into freeze-dried tubes, stored in liquid nitrogen, and then returned to the laboratory for RNA extraction.

### cDNA library preparation

We used the TRIzol reagent (Invitrogen, Carlsbad, CA, USA) and DNase I (Qiagen, Beijing, China) to isolate total RNA. The quality of purified RNA was assessed using 1.5% agarose gel electrophoresis to confirm the absence of genomic DNA, and RNA integrity was estimated using the RNA Nano6000 Assay Kit and a Bioanalyzer 2100 system (Agilent Technologies, Santa Clara, CA, USA). Each sample utilized 3 μg of total RNA (with RNA integrity number values larger than 7.0) for cDNA library construction. Ribosomal RNA (rRNA) was removed using the Epicentre Ribo-Zero rRNA Removal Kit (Epicentre, Madison, WI, USA), and the rRNA-free residue was cleaned by ethanol precipitation. The rRNA-deleted RNA was then used to construct sequencing libraries with a NEBNext Ultra Directional RNA Library Prep Kit for Illumina (NEB, Ipswich, MA, USA), following the manufacturer’s instructions. After fragmentation, first and second strand cDNA synthesis was performed. Adenylation of the 3' ends of DNA fragments was followed by ligation of NEBNext adaptors containing a hairpin loop structure to prepare them for hybridization. The library fragments were then purified using an AMPure XP system (Beckman Coulter, Beverly, MA, USA), with a preference given to cDNA fragments of 150–200 bp in length. Subsequently, the size selected, adaptor ligated cDNA was incubated with the USER enzyme (NEB) at 37 °C for 15 min, followed by 5 min at 95 °C. PCR amplification was then performed using Phusion High-Fidelity DNA polymerase, universal PCR primers, and Index (X) Primer. Finally, the products were purified using the AMPure XP system, and the library quality was assessed using the Agilent Bioanalyzer 2100 system.

### Sequencing and transcriptome assembly

We used the TRIzol reagent (Invitrogen, Carlsbad, CA, USA) and DNase I (Qiagen, Beijing, China) to isolate total RNA. The quality of purified RNA was assessed using 1.5% agarose gel electrophoresis to confirm the absence of genomic DNA. RNA integrity was estimated using the RNA Nano6000 Assay Kit and a Bioanalyzer 2100 system (Agilent Technologies, Santa Clara, CA, USA). Each sample used 3 μg of total RNA (with RNA integrity number values greater than 7.0) for cDNA library construction. Ribosomal RNA (rRNA) was removed using the Epicentre Ribo-Zero rRNA Removal Kit (Epicentre, Madison, WI, USA), and the rRNA-free residue was purified through ethanol precipitation. The rRNA-depleted RNA was then used to construct sequencing libraries using a NEBNext Ultra Directional RNA Library Prep Kit for Illumina (NEB, Ipswich, MA, USA), following the manufacturer's instructions. After fragmentation, first- and second-strand cDNA synthesis was performed. Adenylation of the 3' ends of DNA fragments was followed by ligation of NEBNext adaptors containing a hairpin loop structure to prepare them for hybridization. The library fragments were purified using an AMPure XP system (Beckman Coulter, Beverly, MA, USA), with a preference for cDNA fragments ranging from 150 to 200 bp in length. Subsequently, the size-selected cDNA with ligated adaptors was incubated with the USER enzyme (NEB) at 37 °C for 15 min, followed by 5 min at 95 °C. PCR amplification was then performed using Phusion High-Fidelity DNA polymerase, universal PCR primers, and Index (X) Primer. Finally, the products were purified using the AMPure XP system, and the library quality was assessed using the Agilent Bioanalyzer 2100 system.

### Prediction of the differentially expressed mRNAs and miRNAs

To identify known miRNAs, we first mapped small RNA tags and used miRBase 20.0 as a reference with customized software, miRDeep2^20^, and sRNA-tools-cli. We calculated miRNA counts and base bias using custom scripts for identified miRNA positions of a specific length. To eliminate tags originating from non-miRNA sources such as protein-coding genes, repeat sequences, rRNA, tRNA, snRNA, and snoRNA, we aligned small RNA tags to RepeatMasker, Rfam database, or data specific to the selected species.

To predict new miRNAs, we utilized the properties of miRNA precursor hairpin structures. We utilized miREvo^20^ and miRDeep2^21^ software to analyze the secondary structure, Dicer cleavage site, and minimal free energy of short RNA tags that had not been annotated in previous phases. We used custom scripts to obtain the detected miRNA counts and base bias for identified miRNA positions of a specific length at each position of all identified miRNA.

### Target gene prediction

MiRanda was used to predict the target genes of miRNA in animals. The miRanda algorithm is based on the complementarity of nucleotides (A = U or G≡C). The scoring matrix used for this analysis also accommodates G = U 'wobble' pairs. Complementarity parameters at individual alignment positions are: + 5 for G≡C, + 5 for A = U, + 2 for G = U, and − 3 for all other nucleotide pairs. The algorithm uses affine penalties, which are linear in the length of a gap, for gap opening (− 8) and gap extension (− 2). In addition, after observing known target sites, complementarity scores (both positive and negative values) at the first eleven positions are multiplied by a scaling factor (set at 2.0 in this case) to account for the observed 5'-3' asymmetry. Finally, the following four empirical rules are applied, with positions counted starting at the 5' end of the miRNA: no mismatches at positions 2 to 4; fewer than five mismatches between positions 3–12; at least one mismatch between positions 9 and L-5 (where L is the total alignment length); and fewer than two mismatches in the last five positions of the alignment. With these parameters, the dynamic programming algorithm optimizes the complementarity score between a miRNA sequence and an mRNA sequence (usually a 3' UTR), summed over all aligned positions. It identifies all non-overlapping hybridization alignments in decreasing order of complementarity score down to a specified cutoff value (default value 80)^22^.

### GO and KEGG enrichment analysis

We conducted Gene Ontology (GO) enrichment analysis on the target gene candidates of differentially expressed miRNAs. For Gene Ontology (GO) enrichment analysis, we employed a Wallenius non-central hypergeometric distribution based on GOseq^23^, which can adjust for gene length bias. KEGG^24^ is a database resource for inferring high-level functions and utilities of biological systems such as the cell, organism, and ecosystem from molecular-level data, especially large-scale molecular datasets generated by genome sequencing and other high-throughput experimental technologies (http://www.genome.jp/kegg/). The statistical enrichment of target gene candidates in KEGG pathways was tested using the KOBAS^25^ program.

### Quantitative real-time polymerase chain reaction validation

For the qRT-PCR study, 1 µg of total RNA was reverse transcribed using RT reagent Kits with gDNA Eraser (Takara, Dalian, China) according to the manufacturer’s instructions. The qRT-PCR was conducted using Fast Start Universal SYBR Green Master (ROX) based on routine procedures (Roche, Mannheim, Germany) with a StepOnePlus Real-Time PCR System (Life Technologies, Gaithersburg, MD, USA).

### Ethics statement

The animal study received approval from the Institutional Animal Care and Use Committees of South China Agricultural University (Permit number: 2018-P002). All experimental procedures and sample collection methods adhered to the Regulation on the Administration of Laboratory Animals (CLI.2.293192, 2017 Revision, State Council, China).

## Results

### Read mapping

Before analyzing the data from the three groups of goats, a quality control analysis was conducted. Raw reads of low quality were removed, and the final data were separated into three groups based on age: 0 months (M0-1, M0-2, M0-3), 3 months (M3-1, M3-2, M3-3), and 6 months (M6-1, M6-2, M6-3). The clean reads for each group totaled 12,258,488; 10,654,110; 10,020,864; 10,145,526; 10,475,137; 10,285,628; 11,097,041; 10,663,539; and 10,665,853, respectively. Over 91% of the reads were mapped to the Capra hircus reference genome, and the GC content ranged from 45.56% to 49.30%. The Q30 values for all samples were above 95% (Table 1). These results indicate that the sequencing data was of high quality and is suitable for further analysis.

**Table 1:** Summary of clean reads mapped to the Capra hircus reference genome.

Sample	Total reads	Clean reads	Q30 (%)	GC content (%)	Total mapped
M0-1	12,342,810 (100.00%)	12,258,488 (99.32%)	97.51%	48.83%	85,035,701 (94.17%)
M0-2	10,722,301 (100.00%)	10,654,110 (99.36%)	97.53%	49.30%	93,669,001 (95.9%)
M0-3	10,528,410 (100.00%)	10,020,864 (95.18%)	96.23%	47.06%	92,859,053 (96.4%)
M3-1	10,202,091 (100.00%)	10,145,526 (99.45%)	97.58%	46.56%	86,174,540 (92.84%)
M3-2	10,562,413 (100.00%)	10,475,137 (99.17%)	97.24%	46.93%	103,869,223 (91.09%)
M3-3	10,915,904 (100.00%)	10,285,628 (94.23%)	95.89%	45.46%	100,660,879 (92.63%)
M6-1	11,209,776 (100.00%)	11,097,041 (98.99%)	97.55%	46.22%	85,143,246 (94.45%)
M6-2	11,445,736 (100.00%)	10,663,539 (93.17%)	95.92%	46.73%	100,742,280 (93.31%)
M6-3	10,768,076 (100.00%)	10,665,853 (99.05%)	97.11%	46.83%	106,918,408 (93.71%)

### Enrichment analysis of the differentially expressed mRNAs

**Figure 1:**
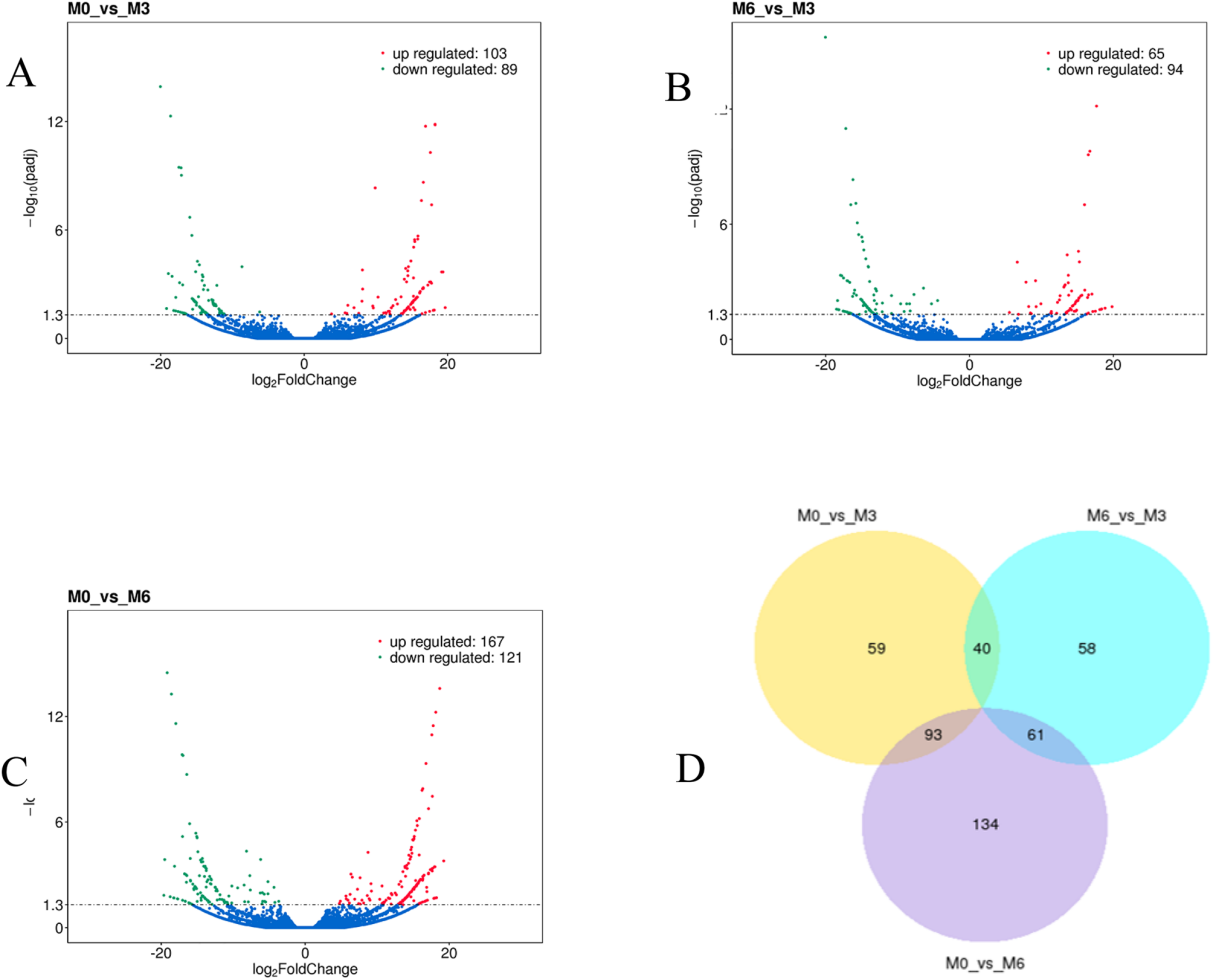
Number of DEGs at different time stage comparisons. The number of up- and downregulated DE-mRNAs across three comparisons: (**A**) M0 vs. M3, (**B**)M0 vs. M6, (**C**)M6 vs. M3, with the threshold set to q < 0.05. (**D**)The venn diagram shows the number of DEGs across three comparisons: M0 vs. M3, M0 vs. M6, M6 vs. M3.

For the identification of differentially expressed genes, we used a significance threshold of p < 0.05, which resulted in the detection of 977 mRNAs. Among these, 77 mRNAs were found to be upregulated, and 75 mRNAs were downregulated in the M3 group compared to the M0 group. The M6 group exhibited 515 upregulated and 402 downregulated mRNAs in comparison to the M0 group. In the comparison between the M3 and M6 groups, we observed 21 upregulated mRNAs and 32 downregulated mRNAs (Fig. 1). To better understand the relationships and similarities between the different libraries, we conducted systematic cluster analyses to analyze the expression patterns of the DEmRNAs. In this article, we cite the research results of Zhao^1^, mainly focusing on mRNA research data. We would like to express our gratitude to Zhao for sharing mRNA-related information with us, as it has laid an important foundation for our research.

**Figure 2:**
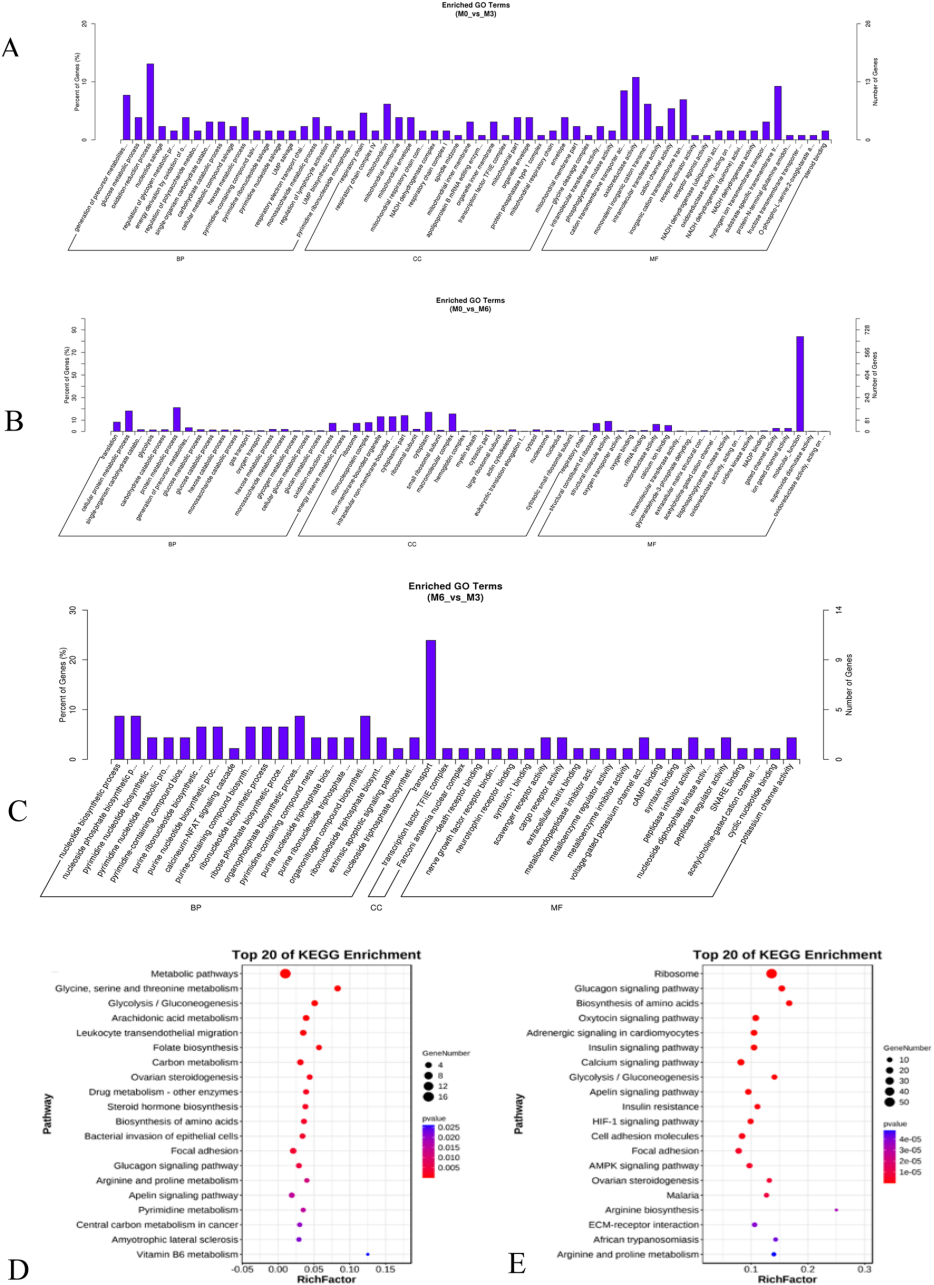
(**A**) Biological process enriched by the differentially expressed mRNAs in M0 vs. M3 group. (B) in M0 vs. M6. (**C**) in M6 vs. M3. (**D**) The top KEGG enrichment analyses of the differentially expressed mRNAs in M0 vs. M3. (**E**) in M0 vs. M6.

In order to understand the functional implications of these mRNAs in regulating litter size, we conducted Gene Ontology (GO) and Kyoto Encyclopedia of Genes and Genomes (KEGG) pathway analyses. We identified a total of 519 significant Gene Ontology (GO) terms with a p-value < 0.05. Among these terms, the M0 vs. M3 group showed significant enrichment in processes related to glucose and glycogen metabolism, generation of precursor metabolites and energy, as well as oxidation–reduction processes (Fig. 2A) (Supplementary Information 1). The differential expression analysis revealed that a significant portion of genes between the M0 and M6 groups were associated with cellular protein metabolic processes, glycolysis, and protein metabolic processes (Fig. 2B) (Supplementary Information 2). The M6 vs. M3 group was mainly enriched in nucleoside and nucleoside phosphate biosynthetic processes, calcineurin-NFAT signaling cascade, and organonitrogen compound biosynthetic processes (Fig. 2C) (Supplementary Information 3). Moreover, within the M0 vs. M3 group, we discovered five statistically significant KEGG pathways (P < 0.05) related to muscle growth and development. These pathways include metabolic pathways, glycine, arachidonic acid metabolism, serine, and threonine metabolism, biosynthesis of amino acids, and focal adhesion (Fig. 2D) (Supplementary Information 4). The M0 vs. M6 group is also enriched in the ribosome, biosynthesis of amino acids, calcium signaling pathway, focal adhesion, and AMPK signaling pathway (Fig. 2E) (Supplementary Information 5). Interestingly, we identified differentially expressed genes (DEGs). One of these genes, OR2AP1 (gene ID: 100861186), was consistently found in all three groups (Supplementary Information 6). In the investigation comparing the M0 and M3 groups, MYL10 emerged as one of the top 50 differentially expressed genes (DEGs) (Supplementary Information 7). Intriguingly, MYL10 demonstrated active involvement in the focal adhesion signaling pathway, indicating its plausible influence on the regulation of skeletal muscle growth and development. In the first 50 DEGs of the M0 vs. M6 group, RYR3 exhibited significant involvement in the calcium signaling pathway (Supplementary Information 7), emphasizing its relevance to cell differentiation and muscle development. Meanwhile, CSRP3 was found to be highly expressed in the first 50 degrees of both M0 vs. M6 and M0 vs. M3 groups. This gene is associated with muscle development, muscle cell maintenance, transcription-related regulation, signal transduction, cytoskeletal organization, and tissue-specific cell growth and development.

### Enrichment analysis of the differentially expressed miRNAs

**Figure 3:**
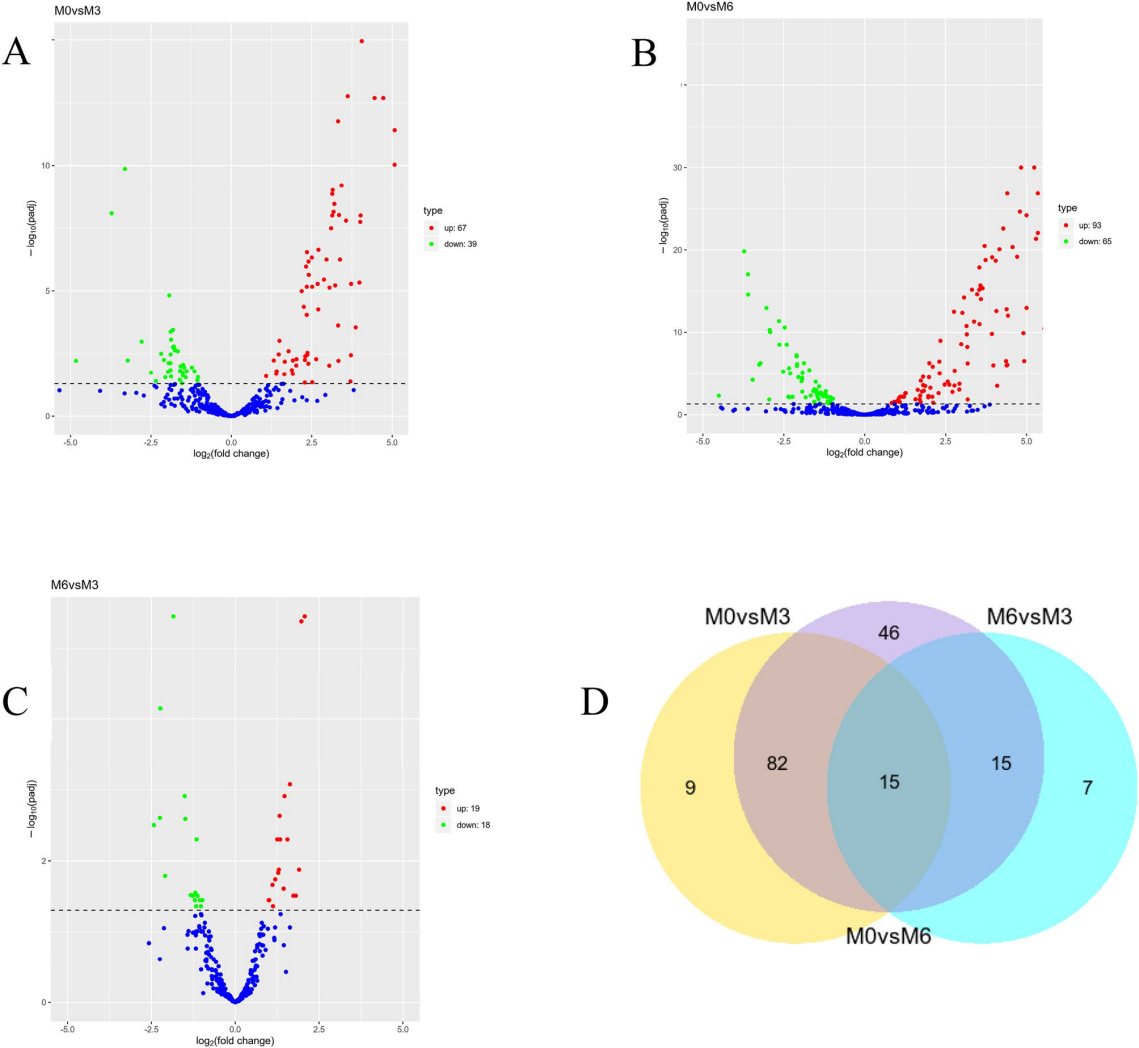
Number of DE-miRNAs at different time stage comparisons. The number of up- and downregulated DE-miRNAs across three comparisons: (**A**) M0 vs. M3, (**B**)M0 vs. M6, (**C**)M6 vs. M3, with the threshold set to q < 0.05. (**D**)The venn diagram shows the number of DE-miRNAs across three comparisons: M0 vs. M3, M0 vs. M6, M6 vs. M3.

Throughout our investigation, we identified alterations in the expression levels of 174 miRNAs (Supplementary Information 9). Out of these, 67 miRNAs showed up-regulation, whereas 39 miRNAs demonstrated down-regulation in the M3 group compared to the M0 group. In a similar manner, we observed 93 miRNAs displaying upregulation and 65 miRNAs showing downregulation in the M0 vs. M6 group comparison. Additionally, in the comparison between the M6 and M3 groups, 19 miRNAs were upregulated, and 18 miRNAs were downregulated (Fig. 3: A–D).

**Figure 4:**
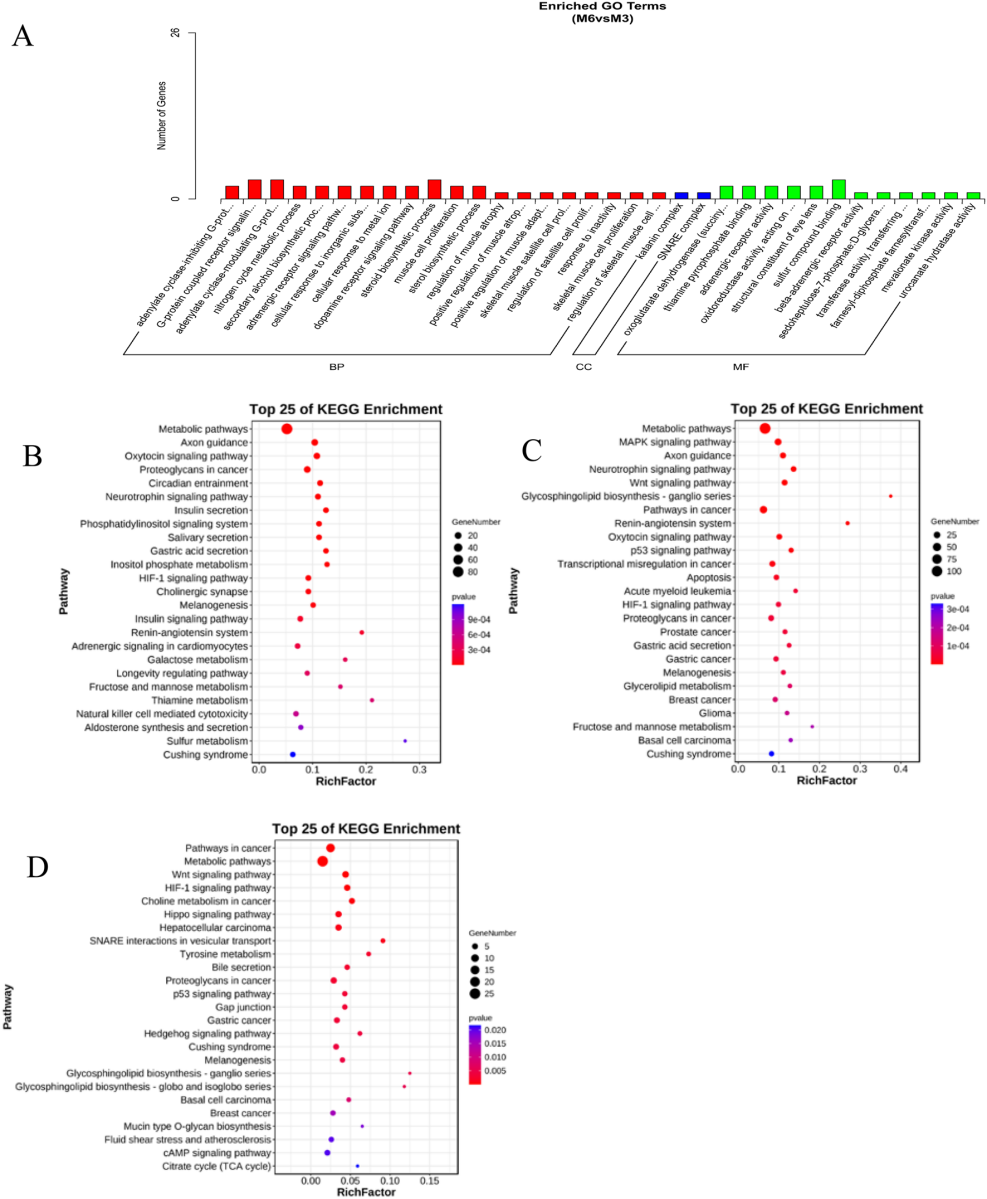
(**A**) Biological process enriched by the DE-miRNAs in M0 vs. M3 group. (**B**) The top KEGG enrichment analyses of the DE-miRNAs in M0 vs. M3. (**C**) in M0 vs. M6. (**D**) in M6 vs. M3.

Further analysis using Gene Ontology (GO) revealed 645 significantly enriched GO terms (P < 0.05). Additionally, we conducted a meticulous examination of GO terms associated with growth and development. We found that the M6 vs. M3 group was mainly enriched in oxidoreductase activity, muscle cell proliferation, skeletal muscle satellite cell proliferation, regulation of satellite cell proliferation, skeletal muscle cell proliferation, and regulation of skeletal muscle cell proliferation (Fig. 4A) (Supplementary Information 10). The KEGG pathway analysis revealed a significant enrichment of differentially expressed miRNAs in various pathways. Specifically, in the M0 vs. M3 group, there was prominent enrichment in pathways such as insulin secretion, insulin signaling, metabolic pathways, and HIF-1 signaling (Fig. 4B) (Supplementary Information 11). The comparison between the M0 and M6 groups displayed enrichment in several pathways, including MAPK signaling, metabolic pathways, Wnt signaling, and HIF-1 signaling (Fig. 4C) (Supplementary Information 12). Finally, the M6 vs. M3 group showed significant enrichment in the Hippo signaling pathway, metabolic pathways, Wnt signaling, and HIF-1 signaling (Fig. 4D) (Supplementary Information 13). Metabolic pathways and the HIF-1 signaling pathway were consistently identified as the major enrichment targets for the differentially expressed miRNAs across all three groups (Supplementary Information 14).

Interestingly, 15 miRNAs were found to be differentially expressed in all three groups. It includes chi-miR-127-3p, chi-miR-133a-3p, chi-miR-193a, chi-miR-193b-3p, chi-miR-29c-5p, chi-miR-365-3p, chi-miR-369-5p, chi-miR-380-3p, chi-miR-381, chi-miR-410-3p, chi-miR-411a-3p, chi-miR-411a-5p, chi-miR-412-5p, chi-miR-493-3p, chi-miR-542-3p. Five of the differentially expressed miRNAs were identified to be specifically involved in muscle growth and development (chi-miR-127-3p, chi-miR-133a-3p, chi-miR-193b-3p, chi-miR-365-3p, chi-miR-381), while six were involved in cell proliferation (chi-miR-193a, chi-miR-29c-5p, chi-miR-369-5p, chi-miR-380-3p, chi-miR-493-3p, chi-miR-542-3p) (Supplementary data). These findings suggest that these miRNAs may play a significant role in muscle growth, development, and cell proliferation processes.

### Prediction and functional analysis of differentially expressed miRNA target genes

**Figure 5:**
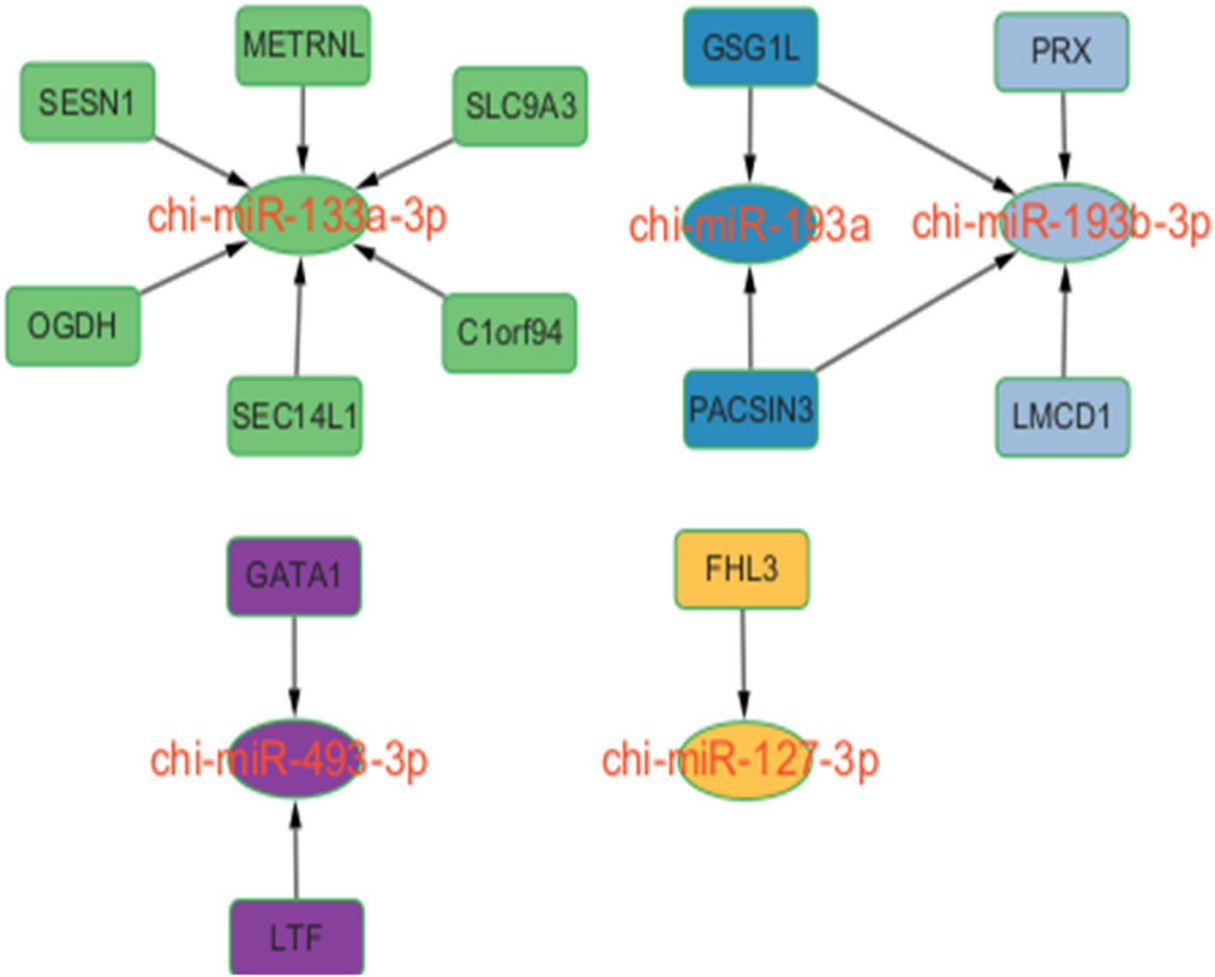
Interaction diagram of differentially expressed miRNA targeted differentially expressed mRNA.

**Figure 6:**
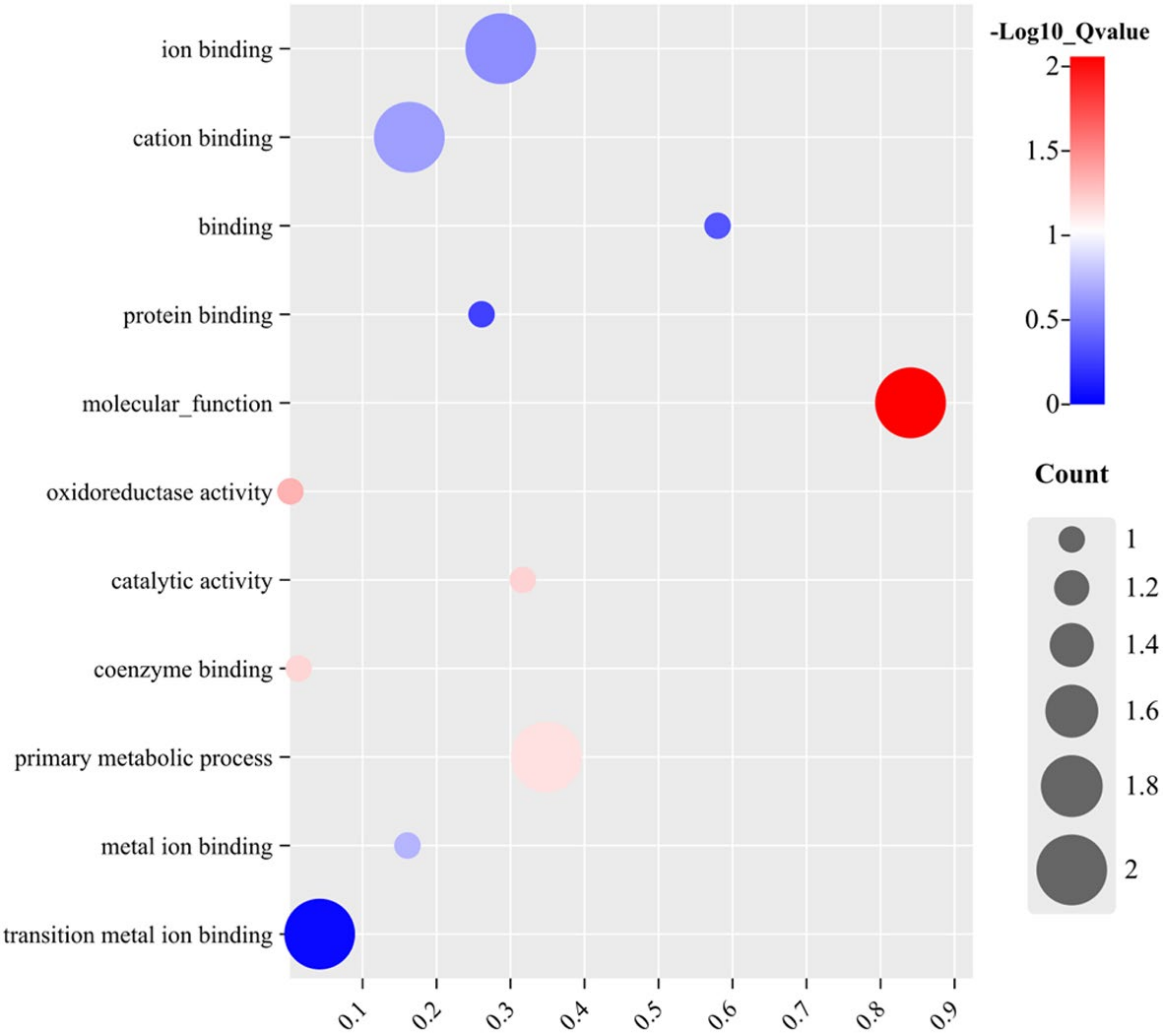
GO enrichment analysis of differentially expressed target genes.

Through miRNA target gene prediction using miRanda, we constructed a prediction of DEmiRNA-mRNA interactions. We identified a total of 162 mRNAs targeting 15 differentially expressed miRNAs in the three groups. Out of these 162 mRNAs, 13 were differentially expressed, and there was minimal overlap in the target genes that were differentially expressed. Among them, there were 15 miRNA-mRNA interaction pairs involving 5 miRNAs and 13 mRNAs (Fig. 5). Functional analyses of these differentially expressed target genes revealed that they were mainly enriched in the binding of various ions, including cations, metal ions, and transition metal ions, as well as in primary metabolic processes, protein binding, and oxidoreductase activity (Fig. 6).

### Gene network validation by quantitative real-time polymerase chain reaction (qRT-PCR)

**Figure 7:**
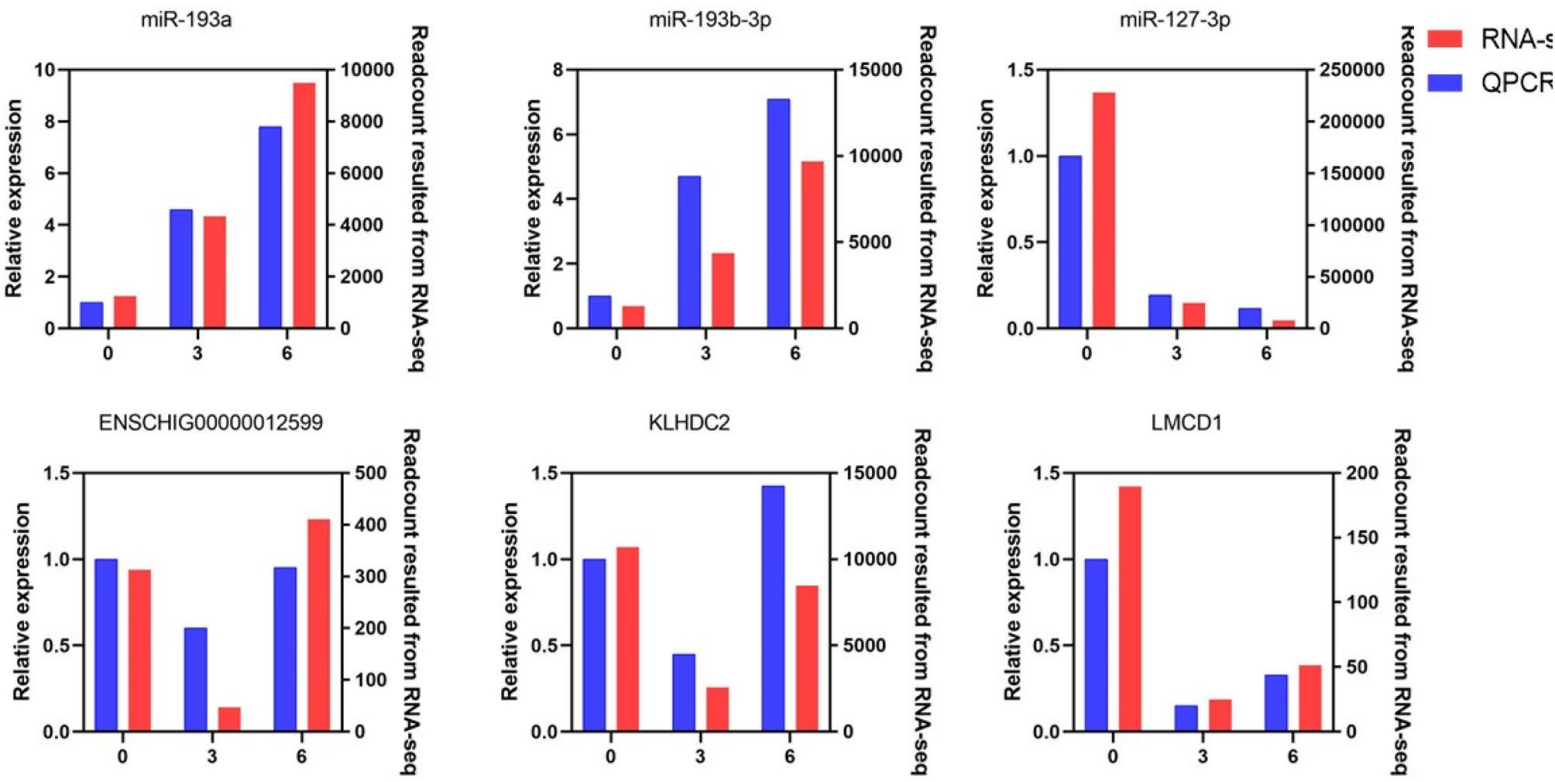
The expression level of three miRNAs (miR-193a、miR-193b-3p、miR-127-3p) and three mRNA (ENSCHIG00000012599、KLHDC2、LMCD1) were validated by qRT-PCR and compared with the results of RNA-SEQ. For qRT-PCR data, miRNA expression was normalized to U6, mRNA expression was normalized to GAPDH.

We randomly selected three differentially expressed miRNAs and three differentially expressed mRNAs for qRT-PCR analysis. Results demonstrated that the gene expression trend in the two experimental groups was consistent between RNA-Seq and qRT-PCR methods, although the fold change of the expression level, to some extent, differed between the two sets of data (Fig. 7). It is proven that the transcriptome sequencing results are reliable and can be used for follow-up studies.

## Discussion

In the past decade, high-throughput sequencing technology has become increasingly widespread in animal science, enabling the discovery of functional genes. This technique has been instrumental in advancing our understanding of miRNAs in studies on muscle growth and development in animals. It has also influenced studies conducted specifically in sheep and goats. MiRNAs, short for microRNAs, are small RNA molecules that function as post-transcriptional regulators by selectively binding to complementary sequences within specific messenger RNAs (mRNAs). This binding often leads to mRNA degradation or translational repression^26^. Studies have shown that miRNAs play a crucial role in the development and differentiation of skeletal muscle, which is a highly coordinated biological process regulated by a network of myogenic transcription factors^27^.

Several studies have identified specific miRNAs that play a role in muscle growth and development in goats. For example, Lyu et al.^28^ discovered that chi-miR-487b-3p regulates goat myoblast growth by targeting IRS1 and affecting the PI3K/Akt signaling pathway, thereby inhibiting skeletal myogenesis. Liu et al.^29^ established that miR-124a functions as a regulatory factor for VMP1 in muscle tissue. Modulating the expression of miR-124a, either through overexpression or inhibition, can exert effects on myoblast proliferation and autophagy. Shen et al.^30^ conducted a comparison of transcriptome profiles in the dorsal longus muscle of Liaoning cashmere goats and Ziwuling black goats. They identified several genes that exhibited differences in skeletal muscle growth and development, such as PEBP1, SPARC, COL3A1, SOCS2, BTG2, and MB. Tripathi AK et al.^31^ found that miR-1, miR-133b, miR-222, and miR-30a are involved in the specificity of muscle development and differentiation. Furthermore, Ling et al.^32^ performed a comprehensive analysis of miRNA in goat fetuses and newborns at seven different stages. They conducted miRNA profiling and identified a total of 421 known miRNAs and 228 novel miRNAs, which provided valuable insights into the temporal expression patterns during skeletal muscle development in goats. These studies provide compelling evidence of the importance of miRNAs in coordinating muscle growth and development.

In order to further investigate the role of miRNAs in muscle growth and development in goats, we conducted transcriptome sequencing and carefully examined the different expression patterns of mRNAs and miRNAs in three distinct groups of Leizhou goats at 0, 3, and 6 months of age. In our study, we identified a number of valuable mRNAs and miRNAs that may play important roles in promoting and influencing muscle growth and development, thus shedding light on the complex mechanisms involved in this process. This is also important for improving the meat production of livestock in production practice. Meanwhile, this will provide a theoretical basis for the future elucidation of the molecular mechanisms of skeletal muscle development in Leizhou goats, and provide new ideas for the molecular breeding and development of Leizhou goats (Supplementary table 1).

Among the three groups, we observed significantly high expression levels of five muscle-specific miRNAs (miR-127-3p, miR-133a-3p, miR-193b-3p, miR-365-3p, miR-381). Situated within the Dlk1-Dio3 region's miRNA cluster, miR-127 has been reported to participate in the development of normal cell lineages, including embryonic stem cells^33^ and fibroblasts^34^. However, it is worth noting that the miR-127 precursor can transcribe two distinct types of mature miRNAs, namely miR-127-3p and miR-127-5p ^35^. In earlier investigations, miR-127-3p was found to be abundantly expressed in skeletal muscle, myocardial muscle of adult mice, and proliferating C2C12 cell lines. Moreover, it was shown to exert regulatory effects on muscle cell proliferation through its interaction with KMT5a^36^. In another study, it was observed that miR-127-3p induces myogenesis through its interaction with Vamp2. By transfecting miR-127-3p into C2C12 cells, researchers demonstrated that miR-127-3p directly targets Vamp2, thereby regulating myoblast proliferation and differentiation^35^. Additionally, it was discovered that miR-127-3p also affects myoblast proliferation by targeting Sept7^37^. In the investigation, we observed a differential expression of FHL3, a target of miR-127-3p, across the 0–6 months age range. Its expression consistently increases from 0 to 6 months. In C2C12 myoblasts, FHL3 was primarily localized in the nucleus. When integrin engagement occurs, it undergoes translocation to actin stress fibers and focal adhesions. Moreover, FHL3's ability to inhibit α-actinin-mediated actin bundling emphasizes its crucial role as a regulator of actin cytoskeletal dynamics in skeletal myoblasts^38^. Additionally, the interaction between FHL3 and MyoD may play a role in regulating MyoD-dependent transcription of muscle genes, thereby contributing to myogenesis^39^. Therefore, we predict that miR-127-3p regulates myogenesis by targeting FHL3. We observed a significant increase in the expression of miR-133a-3p from month 0 to month 6 in DE-miRNAs (p < 0.05). By targeting PRRX1 expression, miR-133a-3p exerts a significant inhibitory effect on myogenic cell proliferation and promotes myogenic cell differentiation^40^. The Akt/mTOR/S6K pathway plays a crucial role in muscle growth and hypertrophy. The reduction of miR-133a-3p expression can stimulate muscle growth through the activation of the Akt/mTOR/S6K signaling^41^. According to our research, SESN1, which is a differentially expressed target gene of miR-133a-3p, is a stress-responsive protein involved in regulating inflammatory responses, muscle atrophy, reactive oxygen species, and oxidative stress^42^. Simultaneously, overexpression of SESN1 significantly increased the proliferation of myogenic cells, inhibited apoptosis, and promoted differentiation^43^. The expression of SESN1 from 0 to 6 months of age showed a tendency to increase and then decrease, which may be related to its involvement in muscle growth and differentiation. Therefore, we hypothesized that the overexpression of SESN1 in Leizhou goats after birth resulted in rapid proliferation of myoblasts and muscle growth, but this trend slowed down with increasing age. In chickens, PPARGC1A promotes intramuscular fatty acid oxidation, drives the conversion of fast-twitch to slow-twitch myofibers, and enhances skeletal muscle mass. Notably, miR-193b-3p is the key regulator that governs PPARGC1A expression^44^. Recent investigations have demonstrated that miR-193b-3p in goats enhances myoblast proliferation by activating insulin-like growth factor-2 mRNA-binding protein through binding to its 3' untranslated region (UTR). In our study, PACSIN3, the gene targeted by miR-193b-3p, is involved in vesicle formation and transport and is predominantly expressed in muscle tissue^45^. Recent studies have shown that PACSIN3 affects the differentiation of primary skeletal muscle cells by interacting with IL-6 in skeletal muscle cells^46^. Meanwhile, we found that another differentially expressed target gene of miR-193b-3p, LMCD1, also appears to be associated with muscle growth and differentiation. It has been found that LMCD1 can act as a gene closely related to skeletal muscle function. It inhibits the expression of muscle regulatory proteins while also increasing muscle protein synthesis and fiber size. LMCD1 is a positive regulator of muscle mass^2^. It has also been found that LMCD1 can enhance cell proliferation, glycolytic capacity, epithelial-mesenchymal transition (EMT), and lower the epigenetic barrier by up-regulating epigenetic factors (EZH2, WDR5, BMI1, and KDM2B) in the early stages of reprogramming, making cells more likely to acquire pluripotency ^3^. Hence, we hypothesized that miR-193b-3p influences the differentiation and proliferation of skeletal muscle cells by targeting PACSIN3 and LMCD1. The expression level of miR-365-3p among differentially expressed miRNAs significantly increased from 0 to 6 months (p < 0.05). MiR-365-3p regulates skeletal muscle development by targeting SelT through muscle development-related factors, mitochondrial biosynthesis, and the calcium ion pathway^47^. In a study on primary bovine myoblast cell proliferation and differentiation, the upregulation of miR-365-3p significantly reduced the expression of cell cycle proteins D1 (CCND1), cyclin-dependent kinase 2 (CDK2), and proliferating cell nuclear antigen (PCNA). This reduction in expression led to the inhibition of cell proliferation but also facilitated myotube formation. It can be hypothesized that skeletal muscle development and muscle tube formation occur in Leizhou goats from 0 to 6 months. Among the miRNAs analyzed, miR-381 exhibited the most significant down-regulation among all the differentially expressed miRNAs in the three groups. In a study focused on the skeletal muscle of Liaoning cashmere goats, researchers observed that miR-381 exhibited the most significant down-regulation among the examined miRNAs. Moreover, miR-381 was found to significantly increase the expression levels of myosin heavy chain (MyHC), myogenin (MyoG), and myocyte enhancer factor 2C (MEF2C) in primary goat skeletal muscle satellite cells (SMSCs). This effect resulted in the promotion of myogenic differentiation in goat skeletal muscle stem cells (SMSCs). Recent studies on miRNAs in sheep skeletal muscle have shown that miR-381 targets several muscle-associated mRNAs, and the genes it targets are implicated in skeletal muscle development^48^. Therefore, miR-381 has the potential to regulate skeletal muscle development by targeting multiple myogenic mRNAs in Leizhou goats. Nevertheless, more research is required to elucidate the underlying mechanism.

Within the group of identified differentially expressed genes (DEGs), MYL10 is a gene that encodes a protein. In livestock and poultry research focused on the molecular mechanisms of meat quality in crossbred sheep, researchers observed the involvement of MYL10 in regulating muscle growth and development. In a study utilizing whole-genome bisulfite sequencing and mRNA sequencing on cloned piglets, the examination of the tongue and biceps femoris muscles revealed that MYL10 plays a critical role in the development of embryos, as well as tissues and organs^49^. We observed a decrease in MYL10 expression in the M0 vs. M3 group. Through bioinformatics analysis, we found that MYL10 is primarily involved in calcium ion binding pathways. The expression of the RyR3 gene was found to be reduced in the M0 vs. M3 group and M0 vs. M6 group. In the adult diaphragm muscle, RyR3 accounts for 1–4% of the total ryanodine binding sites^50^, while in most adult mammalian fast-twitch muscles, it constitutes only 0–0.2% of the total^22,51^. Neonatal mammalian skeletal muscle contains both type 1 and type 3 ryanodine receptors (RyR1 and RyR3) located in the sarcoplasmic reticulum membrane. A study has revealed that RyR3 is strengthened through calcium-induced calcium release (CICR), which leads to strong, uniform, and simultaneous activation of Ca (2 +) release throughout the cell body. This mechanism plays a critical role in Ca (2 +) signaling for cell differentiation and muscle contraction^52^. In mice, during the neonatal stage of diaphragm muscle development (2–15 days after birth), RyR3 was found to be more highly expressed compared to the same muscle in adult mice. This observation indicates a preference for RyR3 expression in differentiated skeletal muscle cells^53^. Functional analysis indicated that RyR3 is involved in the KEGG pathway "Calcium signaling pathway." Based on our findings, we hypothesize that RyR3 plays a role in the skeletal muscle development and differentiation of Leizhou goats. CSRP3, a member of the cysteine-rich protein family, belongs to the group of muscle-specific LIM-only factors and shows specific expression in skeletal muscle. In chicken primary muscle cells, siRNA-mediated CSRP3 knockout demonstrated that silencing CSRP3 resulted in the downregulation of myogenic genes and the upregulation of atrophy-related genes^54^. In livestock and poultry research, the inhibition of CSRP3 through knockdown has been found to suppress chicken satellite cell differentiation by regulating Smad3 phosphorylation in the TGF-β signaling pathway^55^. The expression of CSRP3 mRNA is increased during the developmental stages of skeletal muscle in porcine embryos, suggesting its potential significance in muscle growth^56^. In our investigation, we observed a decrease in the expression of the CSRP3 gene in the M0 vs. M3 group and the M0 vs. M6 group. Consequently, we formulated the hypothesis that CSRP3 might play regulatory roles in the growth and development of Leizhou goats.

## Conclusion

In summary, the complex process of muscle growth and development in goats is regulated by multiple miRNAs and mRNAs working in coordination. Our study revealed differential expression of several miRNAs, namely miR-127-3p, miR-133a-3p, miR-193b-3p, miR-365-3p, miR-38, and their target genes FHL3, SESN1, PACSIN3, LMCD1, along with differential expression of mRNA MYL10, RYR3, and CSRP3 in Leizhou goats at different ages (0–6 months). These findings provide a new reference and theoretical basis for in-depth investigation of the molecular regulatory mechanisms of muscle development in Leizhou goats. In the next study, we will further explore the functions and regulatory mechanisms of key genes, combine transcriptomic data and proteomic data, and carry out the study of protein function and interaction network to comprehensively understand the molecular regulatory network of goat muscle development.

### Supplementary Information

Supplementary Information 1.

Supplementary Information 2.

Supplementary Information 3.

Supplementary Information 4.

Supplementary Information 5.

Supplementary Information 6.

Supplementary Information 7.

Supplementary Information 8.

Supplementary Information 9.

Supplementary Information 10.

Supplementary Information 11.

Supplementary Information 12.

Supplementary Information 13.

Supplementary Information 14.

Supplementary Information 15.

Supplementary Information 16.

Supplementary Information 17.

Supplementary Information 18.

Supplementary Information 19.

Supplementary Information 20.

Supplementary Information 21.

Supplementary Information 22.

Supplementary Legends.
