# Author Correction: Transcriptome analysis of mRNA and miRNA in the development of LeiZhou goat muscles

**DOI:** 10.1038/s41598-024-67345-7

**Published:** 2024-07-25

**Authors:** Junjie Fu, Jie Liu, Xian Zou, Ming Deng, Guangbin Liu, Baoli Sun, Yongqing Guo, Dewu Liu, Yaokun Li

**Affiliations:** 1 College of Animal Science, South China Agricultural University, Guangzhou, 510642 China; 2 State Key Laboratory of Livestock and Poultry Breeding, Guangdong Key Laboratory of Animal Breeding and Nutrition, Institute of Animal Science, Guangdong Academy of Agricultural Sciences, Guangzhou, 510640 China; 3 National Local Joint Engineering Research Center of Livestock and Poultry, South China Agricultural University, Guangzhou, 510642 China

Correction to: *Scientific Reports* 10.1038/s41598-024-60521-9, published online 29 April 2024

The original version of this article contained an error in Supplementary Information files 2, 3, 6, 7, 10–12, 14–17 and 21, where column C was incorrectly loaded.

Additionally, in the Results, under subheading ‘Read mapping’, Supplementary files citations were missing from the text.

Finally, Supplementary legends file was omitted.

The original Article and accompanying Supplementary Information files have been corrected.

